# The association between cognitive ability and opioid prescribing in vulnerable older adults with chronic pain in ambulatory care: a secondary data analysis using the Medical Expenditure Panel Survey

**DOI:** 10.1186/s12916-023-03133-w

**Published:** 2023-11-16

**Authors:** Ulrike Muench, Kyung Mi Kim, Zachary Zimmer, Todd B. Monroe

**Affiliations:** 1 Department of Social and Behavioral Sciences, School of Nursing, University of California, 490 Illinois St., Floor 12, Box 0612, San FranciscoSan Francisco, CA 94143 USA; 2 Philip R. Lee Institute for Health Policy Studies, School of Medicine, University of California, San Francisco, USA; 3 Healthforce Center, University of California, San Francisco, San Francisco, USA; 4 Office of Research Patient Care Services, Stanford Health Care, Stanford, USA; 5 Clinical Excellence Research Center, School of Medicine, Stanford University, Stanford, USA; 6 Global Aging and Community Initiative and Department of Family Studies & Gerontology, Mount Saint Vincent University, Halifax, Canada; 7 Center for Healthy Aging Self-Management, and Complex Care, College of Nursing, The Ohio State University, Columbus, USA

**Keywords:** Pain, Dementia, Alzheimer’s disease and Alzheimer’s disease and related dementia, Chronic pain, Health services research, Medical Expenditure Panel Survey, Primary care

## Abstract

**Background:**

Vulnerable older adults living with Alzheimer’s disease or Alzheimer’s disease and related dementia (AD/ADRD) and chronic pain generally receive fewer pain medications than individuals without AD/ADRD, especially in nursing homes. Little is known about pain management in older adults with AD/ADRD in the community. The aim of the study was to examine opioid prescribing patterns in individuals with chronic pain by levels of cognitive ability in ambulatory care.

**Methods:**

We used the Medical Expenditure Panel Survey (MEPS), years 2002–2017, and identified three levels of cognitive impairment: no cognitive impairment (NCI), individuals reporting cognitive impairment (CI) without an AD/ADRD diagnosis, and individuals with a diagnosis of AD/ADRD. We examined any receipt of an opioid prescription and the number of opioid prescriptions using a logistic and negative binomial regression adjusting for sociodemographic and health characteristics and stratifying by three types of chronic pain (any chronic pain, severe chronic pain, and chronic pain identified through ICD 9/10 chronic pain diagnoses).

**Results:**

Among people with any chronic pain, adjusted odds of receiving an opioid for people with CI (OR 1.41, 95% confidence interval 1.31–1.52) and AD/ADRD (OR 1.23, 95% confidence interval 1.04–1.45) were higher compared to NCI. Among people with chronic pain ICD 9/10 conditions, the odds of receiving an opioid were also higher for those with CI (OR 1.43, 95% confidence interval 1.34–1.56) and AD/ADRD (OR 1.48, 95% confidence interval 1.23–1.78) compared to NCI. Among those with severe chronic pain, people with CI were more likely to receive an opioid (OR 1.17, 95% confidence interval 1.07–1.27) relative to NCI (OR 0.89, 95% confidence interval 0.75–1.06). People with AD/ADRD experiencing severe chronic pain were not more likely to receive an opioid compared to the NCI group. Adjusted predicted counts of opioid prescriptions showed more opioids in CI and AD/ADRD in all chronic pain cohorts, with the largest numbers of opioid prescriptions in the severe chronic pain and ICD 9/10 diagnoses groups.

**Conclusions:**

The results suggest increased opioid use in people living with CI and AD/ADRD in the ambulatory care setting and potentially indicate that these individuals either require more analgesics or that opioids may be overprescribed. Further research is needed to examine pain management in this vulnerable population.

## Background

Globally, it is estimated that ~ 57 million people live with Alzheimer’s disease or Alzheimer’s disease and related dementia (AD/ADRD) [1] and that ~ 1.5 billion people suffer from chronic pain [2], making both AD/ADRD and chronic pain among the most important public health problems in the world today. AD/ADRD and chronic pain frequently coexist [3, 4]. Among people with AD/ADRD, 50–80% are estimated to experience chronic pain, depending on the severity of cognitive impairment, setting, and study design [5–7]. The management of chronic pain in people with AD/ADRD is complex, because the behavioral symptoms of AD/ADRD can mimic pain, and as the disease progresses, verbal communication diminishes, which makes accurately calibrating treatment difficult for clinicians. It is a well-established problem that these and other factors generally lead to undertreatment of pain in people with AD/ADRD [8] despite emerging evidence that individuals with subtypes of dementia, such as vascular dementia and dementia with Lewy bodies, may have increased pain perception due to neuropathological changes of the disease in the central nervous system [9], increasing the vulnerability of these older adults with chronic pain.

In nursing homes in particular, undertreatment of pain for people with AD/ADRD is a serious concern [10–14], while in the primary care setting, fewer studies exist and findings are mixed, with some reporting undertreatment [15] and others reporting greater use of opioid and non-opioid analgesics [15, 16]. Given that the high use of opioids in the ambulatory care setting [17, 18] has been recognized as a contributing factor in the US opioid epidemic, it is possible that treatment of chronic pain for people with cognitive impairment living in the community is quite different from the residential care setting. The overall goal of this analysis was to examine opioid prescribing patterns among community-dwelling older adults with chronic pain across levels of cognitive ability. Using nationally representative data, we compared opioid analgesic use in three groups of individuals, those with no cognitive impairment (NCI), those with cognitive impairment (CI) without a diagnosis of AD/ADRD, and those with an AD/ADRD diagnosis.

## Methods

### Data and study design

We used the Medical Expenditure Panel Survey (MEPS), the largest household survey on health service use for the noninstitutionalized US population. The MEPS provides public use files (PUF) [19]. Our analysis, however, included 5-digit diagnosis codes that are not available in the PUFs, and we obtained permission to access these data through the Agency for Healthcare Research and Quality data center. The MEPS yields nationally representative samples and allows for individuals to be followed over a 2-year period with five waves of data collection. This allows researchers to use the panel level data to generate measures that have a temporal component, such as pain lasting over a specific timeframe (see details on chronic pain below), and at the same time allows to merge annual cross-sections. We used data from the years 2002–2017 for a pooled cross-sectional analysis, linking patient demographics, medical conditions, and prescription data. MEPS participants are asked for the name of any prescription medications taken during the calendar year and what pharmacy it was purchased from. Data is then obtained from respective pharmacies with the date when the prescription was filled or refilled, creating a record for each individual medication purchased during the year. We can thus assess the number of times a prescription was purchased for the same, or a different, opioid. Because the medication dose and the number of days supplied on the prescription were only added to the MEPS in later years, we were unable to account for the dose or duration of an opioid in our analysis.

### Measures

Our exposure was a three-level cognitive status variable generated from MEPS responses and AD/ADRD diagnoses: AD/ADRD, CI, and NCI. People with AD/ADRD were identified as those with an AD/ADRD diagnosis. If participants reported experiencing confusion or memory loss, problems making decisions, or requiring supervision but did not have an AD/ADRD diagnosis, they were classified as having CI. If they had neither, they were categorized as NCI.

Our analysis included two outcomes. We measured the receipt of any opioid prescription with a binary yes/no variable and measured the number of opioid prescriptions with a count variable.

### Sample

We included individuals ages 50 and older who experienced chronic pain. We identified chronic pain in three ways: any chronic pain, severe chronic pain, and chronic pain from ICD-9/10 diagnoses codes. We used the second and fourth rounds of MEPS, spanning approximately 6 to 9 months, to identify both any chronic pain and severe chronic pain that lasted for at least 6 months, in line with the clinically accepted definition of chronic pain. Any chronic pain was pain interference reported during the last 4 weeks with normal work outside the home and housework of *a little bit*, *moderately*, *quite a bit*, or *extremely*. Severe chronic pain included reports of pain interference of *quite a bit* or *extreme*. We required both types of pain measures to be reported in both MEPS rounds. Finally, we used ICD-9/10 codes for chronic pain diagnoses reported in previous literature [20]. We excluded individuals requiring a proxy to complete the survey. Excluding individuals who used a proxy to help fill out the MEPS survey meant that our sample consisted of people who likely did not have advanced cognitive impairment.

### Statistical analysis

We conducted summary descriptive analyses for individuals reporting any chronic pain for key study variables across levels of cognitive ability (NCI, CI, AD/ADRD), assessing the differences in baseline characteristics using standardized means [21]. We then examined the likelihood of receiving an opioid using multivariable logistic regression and estimated the number of opioid prescriptions using a negative binomial regression. Regression analyses adjusted for age, gender, race/ethnicity, marital status, education, income, health insurance, Elixhauser Comorbidity Index, depression, rural/urban status, region, and survey year and were stratified by any chronic pain, severe chronic pain, and chronic pain measured with ICD-9/10 codes. We applied the MEPS survey weight to all analyses to account for the MEPS complex survey design.

## Results

Table 1 shows the descriptive statistics for people with any chronic pain across levels of cognitive ability (NCI, CI, AD/ADRD). On average, people with CI and people with AD/ADRD were older than those with NCI (66 years old and 79 years old vs. 64 years old, respectively). Chronic pain was more prevalent in females versus males across all levels of cognitive ability, with approximately two-thirds in the CI and AD/ADRD groups (60.0% and 66.0%) and roughly half in the NCI group (56.3%). Fewer people in the CI and AD/ADRD groups had a college education (17.1% and 13.1%, respectively) compared to people with NCI (24.9%). Approximately, half of those with CI and AD/ADRD (54.9% and 57.8%, respectively) had public insurance. In comparison, roughly a quarter with NCI (26.4%) had public insurance, and the majority had private insurance (67.9%). Finally, people with CI and people with AD/ADRD were more likely to have three or more comorbidities (12.8% and 11.4%, respectively) compared with their NCI counterparts (5.5%).

**Table 1:** Summary characteristics of people with any self-reported chronic pain (*n* = 60,594^a^), by levels of cognitive ability

	Cognitive ability			Standardized mean difference^b^
	No cognitive impairment (NCI) (*n* = 52,854) (*n*/%)^a^	Cognitive impairment (CI) (*n* = 6,381) (*n*/%)^a^	AD/ADRD (*n* = 1,359) (*n*/%)^a^	
Race/ethnicity				0.12
Asian	2563 (3.3)	269 (3.2)	46 (2.6)	
Black	9152 (9.6)	1509 (14.0)	304 (13.6)	
Latine	8144 (8.0)	1065 (10.1)	278 (10.5)	
Others^b^	1162 (2.2)	215 (3.3)	38 (3.2)	
White	31,833 (76.9)	3323 (69.6)	693 (70.1)	
Sex				0.19
Female	30,911 (56.3)	3963 (59.6)	912 (66.0)	
Male	21,943 (43.8)	2418 (40.4)	447 (34.1)	
Marital status				0.80
Married	30,038 (60.6)	2073 (35.5)	457 (37.3)	
Divorced/widowed	18,797 (32.9)	3547 (54.1)	833 (58.9)	
Never married	4019 (6.5)	761 (10.4)	69 (3.8)	
Education				0.32
Less or equal to high school	37,607 (65.6)	5280 (78.5)	1181 (80.8)	
College/bachelor	11,195 (24.9)	881 (17.1)	130 (13.1)	
Masters/doctorate	4052 (9.5)	220 (4.4)	48 (6.1)	
Insurance				0.79
Private	31,877 (67.9)	2065 (40.6)	454 (42.1)	
Public	17,060 (26.4)	4002 (54.9)	902 (57.8)	
Uninsured	3917 (5.8)	314 (4.5)	< 5 (0.1)	
Depression				0.53
Yes	8286 (16.3)	2254 (36.9)	306 (22.7)	
No	44,568 (83.7)	4127 (63.1)	1053 (77.3)	
Elixhauser Comorbidity Index, *n* (%)				0.33
0	18,073 (35.3)	1618 (25.9)	310 (25.1)	
1	22,739 (42.2)	2439 (37.4)	551 (39.0)	
2	9129 (17.1)	1521 (23.9)	342 (24.5)	
≥ 3	2913 (5.5)	803 (12.8)	156 (11.4)	
Rurality				0.11
Urban	43,092 (81.4)	5047 (79.0)	1026 (77.3)	
Rural	9762 (18.6)	1334 (21.0)	333 (22.7)	
Region				0.07
Northeast	8384 (17.9)	1026 (17.6)	173 (15.7)	
Midwest	11,408 (23.6)	1243 (21.4)	259 (22.3)	
South	20,733 (37.2)	2651 (38.7)	633 (40.4)	
West	12,329 (21.4)	1461 (22.4)	294 (21.7)	
Age, years, mean (SD)	63.9 (9.6)	65.7 (11.6)	79.2 (7.5)	2.05
Income, $, mean (SD)	$47,511.1 (45,374.6)	$25,844.9 (29,460.6)	$27,432.0 (26,285.5)	0.56

^a^Frequencies are unweighted, and % are weighted

^b^The reported standardized mean difference for each variable is the maximum of all three pairwise standardized mean difference. Standardized mean differences between 0.2 and < 0.5, 0.5 and 0.8, and > 0.8 are considered small, medium, and large, respectively

The adjusted odds of receiving an opioid were higher for people with CI and AD/ADRD compared to NCI in any chronic pain (CI: OR 1.409, 95% confidence interval 1.306–1.519; AD/ADRD: OR 1.229, 95% confidence interval 1.039–1.454) and chronic pain based on ICD-9/10 diagnoses codes (CI: OR 1.453, 95% confidence interval 1.336–1.580; AD/ADRD: OR 1.477, 95% confidence interval 1.229–1.776). For individuals with severe chronic pain, people with CI were more likely to receive an opioid compared to people with NCI (OR 1.167, 95% confidence interval 1.074–1.269). In contrast, people living with AD/ADRD who experienced severe chronic pain were the only group with no statistically significant differences in receiving an opioid compared with people with NCI (OR 1.132, 95% confidence interval 0.937–1.367). Table 2 shows results by type of chronic pain.

**Table 2:** Unadjusted and adjusted OR with 95% CI for receipt of opioid, by chronic pain type^a^^,b,c,d^

	**Any chronic pain (*n* = 60,594), odds ratio (95% CI)**	**Severe chronic pain (*n* = 36,996), odds ratio (95% CI)**	**Chronic pain ICD 9/10 diagnoses**^**e**^** (*n* = 48,273), odds ratio (95% CI)**
**Unadjusted**			
Cognitive status			
No cognitive impairment (NCI)	1 [reference]	1 [reference]	1 [reference]
Cognitive impairment (CI)	1.876 (1.756–2.005)	1.392 (1.294–1.498)	1.987 (1.845–2.139)
AD/ADRD	1.160 (0.991–1.358)	0.890 (0.748–1.059)	1.482 (1.251–1.757)
**Adjusted**			
Cognitive status			
No cognitive impairment (NCI)	1 [reference]	1 [reference]	1 [reference]
Cognitive impairment (CI)	1.409 (1.306–1.519)	1.167 (1.074–1.269)	1.453 (1.336–1.580)
AD/ADRD	1.229 (1.039–1.454)	1.132 (0.937–1.367)	1.477 (1.229–1.776)
Age	0.978 (0.976–0.981)	0.973 (0.970–0.976)	0.980 (0.977–0.982)
Race/ethnicity			
White	1 [reference]	1 [reference]	1 [reference]
Asian	0.383 (0.328–0.447)	0.341 (0.278–0.417)	0.378 (0.316–0.453)
Black	0.896 (0.840–0.955)	0.831 (0.770–0.898)	0.944 (0.879–1.013)
Latine	0.651 (0.602–0.703)	0.601 (0.547–0.659)	0.658 (0.603–0.717)
Others	1.267 (1.094–1.468)	1.135 (0.954–1.350)	1.124 (0.958–1.319)
Sex			
Female	1 [reference]	1 [reference]	1 [reference]
Male	0.948 (0.901–0.998)	0.896 (0.840–0.955)	1.061 (1.002–1.123)
Marital status			
Married	1 [reference]	1 [reference]	1 [reference]
Divorced/widowed	1.095 (1.036–1.157)	0.992 (0.928–1.061)	1.053 (0.991–1.118)
Never married	0.918 (0.831–1.015)	0.871 (0.769–0.987)	0.893 (0.800–0.998)
Income	0.942 (0.920–0.965)	0.973 (0.943–1.003)	0.904 (0.881–0.927)
Education			
Less or equal to high school	1 [reference]	1 [reference]	1 [reference]
College/bachelor	0.883 (0.829–0.941)	0.948 (0.874–1.029)	0.921 (0.860–0.987)
Masters/doctorate	0.805 (0.727–0.893)	0.888 (0.770–1.025)	0.747 (0.669–0.834)
Insurance			
Private	1 [reference]	1 [reference]	1 [reference]
Public	1.295 (1.221–1.374)	1.188 (1.107–1.274)	1.310 (1.228–1.398)
Uninsured	0.743 (0.663–0.833)	0.691 (0.602–0.793)	0.775 (0.680–0.884)
Depression	1.503 (1.415–1.596)	1.432 (1.333–1.539)	1.518 (1.421–1.622)
Elixhauser Comorbidity Index			
0	1 [reference]	1 [reference]	1 [reference]
1	1.218 (1.146–1.294)	1.158 (1.072–1.250)	1.396 (1.305–1.493)
2	1.635 (1.523–1.756)	1.495 (1.369–1.632)	1.814 (1.678–1.962)
≥ 3	2.339 (2.123–2.577)	2.060 (1.839–2.308)	2.574 (2.318–2.858)
Rurality			
Urban	1 [reference]	1 [reference]	1 [reference]
Rural	1.030 (0.966–1.098)	1.002 (0.927–1.082)	1.070 (0.997–1.149)
Region			
Northeast	1 [reference]	1 [reference]	1 [reference]
Midwest	1.396 (1.281–1.521)	1.427 (1.284–1.587)	1.309 (1.194–1.435)
South	1.470 (1.357–1.593)	1.456 (1.321–1.605)	1.481 (1.359–1.614)
West	1.517 (1.389–1.656)	1.527 (1.371–1.700)	1.479 (1.347–1.625)

^a^Analyses applied survey weights to adjust for MEPS complex survey design

^b^In addition to the variables indicated above, models included region and survey year

^c^Individuals with diagnoses of cancer or opioid use disorder, prescriptions for methadone or buprenorphine, presence of proxy responses, or missing values for cognitive status or pain variables were excluded

^d^Opioids included prescriptions for codeine, hydrocodone, hydromorphone, levorphanol, meperidine, morphine, opium, oxycodone, oxymorphone, pentazocine, propoxyphene, tapentadol, butorphanol, fentanyl, nalbuphine, sufentanil, tramadol, and dihydrocodeine

^e^Chronic pain conditions were identified based on International Classification of Disease (ICD) 9/10 codes following the approach outlined by Mikoz and colleagues [20]

**Fig. 1:**
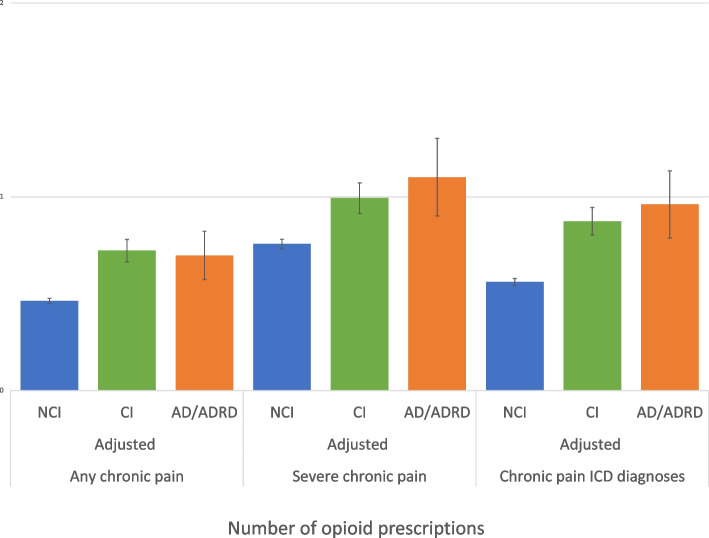
Adjusted predicted numbers of opioid prescriptions by cognitive status, stratified by chronic pain type ^a^Abbreviations: NCI, no cognitive impairment; CI, cognitive impairment; AD/ADRD, Alzheimer’s disease and Alzheimer’s disease and related dementias. ^b^The predicted number of opioid prescriptions were statistically significantly higher for people with CI compared to people with NCI and for people with AD/ADRD compared to people with NCI in the any chronic pain, severe chronic pain, and ICD-9/10 chronic pain diagnoses groups. The model adjusted for age, gender, race/ethnicity, marital status, education, income, health insurance, Elixhauser Comorbidity Index, depression, rural/urban status, region, and survey year and used MEPS complex survey weights. Statistically significant differences compared to NCI (reference group) are indicated with * (all *p*-values *p*  < 0.001). ^c^Individuals with diagnoses of cancer or opioid use disorder, prescriptions for methadone or buprenorphine, presence of proxy responses, or missing values for cognitive status or pain variables were excluded

Adjusted predicted counts of opioid prescriptions for NCI, CI, and AD/ADRD are plotted in Fig. 1. Compared to people with NCI, the predicted number of opioids from the fully adjusted model was higher in CI and AD/ADRD in all chronic pain cohorts and were highest for people living with AD/ADRD in both the severe chronic pain and ICD-9/10 chronic pain groups.

## Discussion

The results from this study support previous research reporting increased opioid use in people with CI and AD/ADRD capable of self-reporting in the primary care setting [16]. Our study cannot ascertain whether these opioid prescribing patterns indicate a clinical need or potential overprescribing. For several decades, studies have reported undertreatment of pain in people living with AD/ADRD. In the 1990s, studies by Ferrell and colleagues [22–24] demonstrated pain assessment challenges leading to undertreatment in nursing home residents with AD/ADRD. This work sparked numerous additional studies demonstrating that potentially poorly managed pain in AD/ADRD was also present in numerous other settings including the community [25], acute care [26], oncology [27], and hospice [28]. In addition, this early work led to the development of numerous observational pain scales for use in people living with severe AD/ADRD alongside several expert panel and professional organization statements on recommendations for improving pain care in AD/ADRD [29–32]. Recently, it has been postulated that the years of research and attention focused on undertreatment or poorly managed pain in AD/ADRD may have led clinicians to become more liberal in prescribing analgesics, including opioids, in people living with AD/ADRD in some settings [33, 34].

Another explanation might be that people living with AD/ADRD require more analgesics due to increased pain sensitivity from neuropathological changes impacting pain regions in the brain. A recent functional neuroimaging study reported that in response to experimental pain, and when compared to cognitively normal controls, people with mild to moderate AD/ADRD verbally reported more pain and displayed greater brain activation in the periaqueductal gray, a key brain region responsible for opioid modulation [33]. Similarly, emerging evidence suggests that individuals with vascular dementia may experience heightened pain affect or pain unpleasantness [35], and dementia with Lewy bodies has been associated with increased pain sensitivity [9]. This would suggest that some populations in our sample who are being prescribed opioids might require higher levels of opioids to achieve similar analgesia relative to cognitively healthy adults, and it is plausible that our findings of people with CI and AD/ADRD receiving more opioid prescriptions in each chronic pain cohort reflect the need for more opioid to achieve adequate analgesia.

Another explanation could be that primary care providers are more likely to prescribe an opioid due to the clinical complexities of distinguishing pain symptoms from behavioral symptoms related to dementia. However, our results demonstrating that people living with AD/ADRD and experiencing severe pain—a population that may need more opioids to receive similar analgesia—were no more likely to receive an opioid compared to people without cognitive impairment. This is potentially an important concern for clinicians who manage pain in people living with AD/ADRD, since this may be a population that requires higher levels of analgesia.

Finally, the results of our analysis could in part be explained by opioids negatively impacting cognition. However, research on this hypothesis is mixed, with findings showing opioids to be both protective [36] and possibly harmful [37, 38] on cognition. The cross-sectional data used in this study do not provide details about the duration of cognitive impairment or opioid use. As a result, we could not determine the sequence of events between cognitive status and opioid use. The interplay between cognition, pain, and opioid use is complex, necessitating further studies to clarify these relationships and pathways. More research is needed to determine the impact of therapeutic short- and long-term exposure to opioids on risk for cognitive impairment and potential development of AD/ADRD as well as further investigation on the role of opioid receptor subtypes as therapeutic targets for future treatments [39].

AD/ADRD and chronic pain are growing population health problems worldwide [40, 41], and chronic pain is a common comorbidity for people living with dementia [4]. Because research has demonstrated alterations in pain reporting among people even with mild dementia [42], and to better understand and improve pain management outcomes in these vulnerable adults, it is critical to examine opioid, and non-opioid, pain medication prescribing at different stages of disease progression. This should include people living with AD/ADRD who have lost the ability to verbally communicate living in both the community and residential settings since managing pain in this population provides additional complexities that may increase the risk of inequities in pain treatment.

Study limitations include the use of survey data that are subject to reporting biases and lack of information regarding the dose and duration of the prescription. Additional data limitations include that we are unable to ascertain the sequencing of cognitive ability and opioid prescriptions within a given survey year. Other limitations include that our analysis examined only opioids and did not analyze or account for other pain-adjuvant medications. It is possible, for example, that individuals with AD/ADRD were more likely to receive behavioral health pain-adjuvant medications such as gabapentin. However, this would further call into questions the higher opioid levels observed in people living with AD/ADRD compared to people with NCI. Furthermore, our measure of cognitive impairment was obtained through self-report and therefore assess the subjective experience of cognitive decline. Studies have reported that while subjective cognitive decline is a predictor of developing AD/ADRD [43, 44], it can also indicate non-neurogenerative disease, such as depression and anxiety [45]. Since this analysis excluded individuals who required a proxy to complete the MEPS, our results cannot be generalized to individuals with advanced AD/ADRD. Ensuring the validity and reliability of survey responses in advanced cognitive impairment is challenging, and likewise, differences between self- and proxy-reported answers potentially present response biases. Finally, it was beyond the scope of this analysis to examine if subtypes of dementia were associated with analgesic medications received.

Strengths of this study are the use of nationally representative data, the inclusion of communicative people living with different stages of cognitive impairment, and the use of multiple chronic pain measures from questionnaires and diagnosis code data that show the consistency of our results across pain measures and support the robustness of our findings.

## Conclusions

Our results highlight the need for evidence-based pain treatment across mild to moderate levels of cognitive impairment and AD/ADRD so that pain management recommendations are available to guide clinical practice. Because pain treatment practices are continuing to evolve in people living with AD/ADRD, and because managing pain and behavioral symptoms in people with cognitive impairment is challenging for clinicians, further research focusing on pain prevalence, pain assessment, and pain management in people across the cognitive spectrum and by dementia subtype is urgently needed to help improve quality of life in people living with cognitive impairment or AD/ADRD.

## Data Availability

The data that support the findings of this study are available from the Agency for Healthcare Research and Quality (AHRQ) [46], but restrictions apply to the availability of these data, which were used under license for the current study, and so are not publicly available. Researchers can apply for the restricted data with AHRQ.

## Abbreviations

AD/ADRD: Alzheimer’s disease and Alzheimer’s disease and related dementia
CI: Cognitive impairment
ICD: International Classification of Diseases
MEPS: Medical Expenditure Panel Survey
NCI: No cognitive impairment
OR: Odds ratio
